# Supplementary figures and images for: Ultra-fast genetic colocalisation across millions of association signals
Source: PLoS Genet. 2026 Jun 17;22(6):e1012209. doi: 10.1371/journal.pgen.1012209 (PMC13289940; doi:10.1371/journal.pgen.1012209)

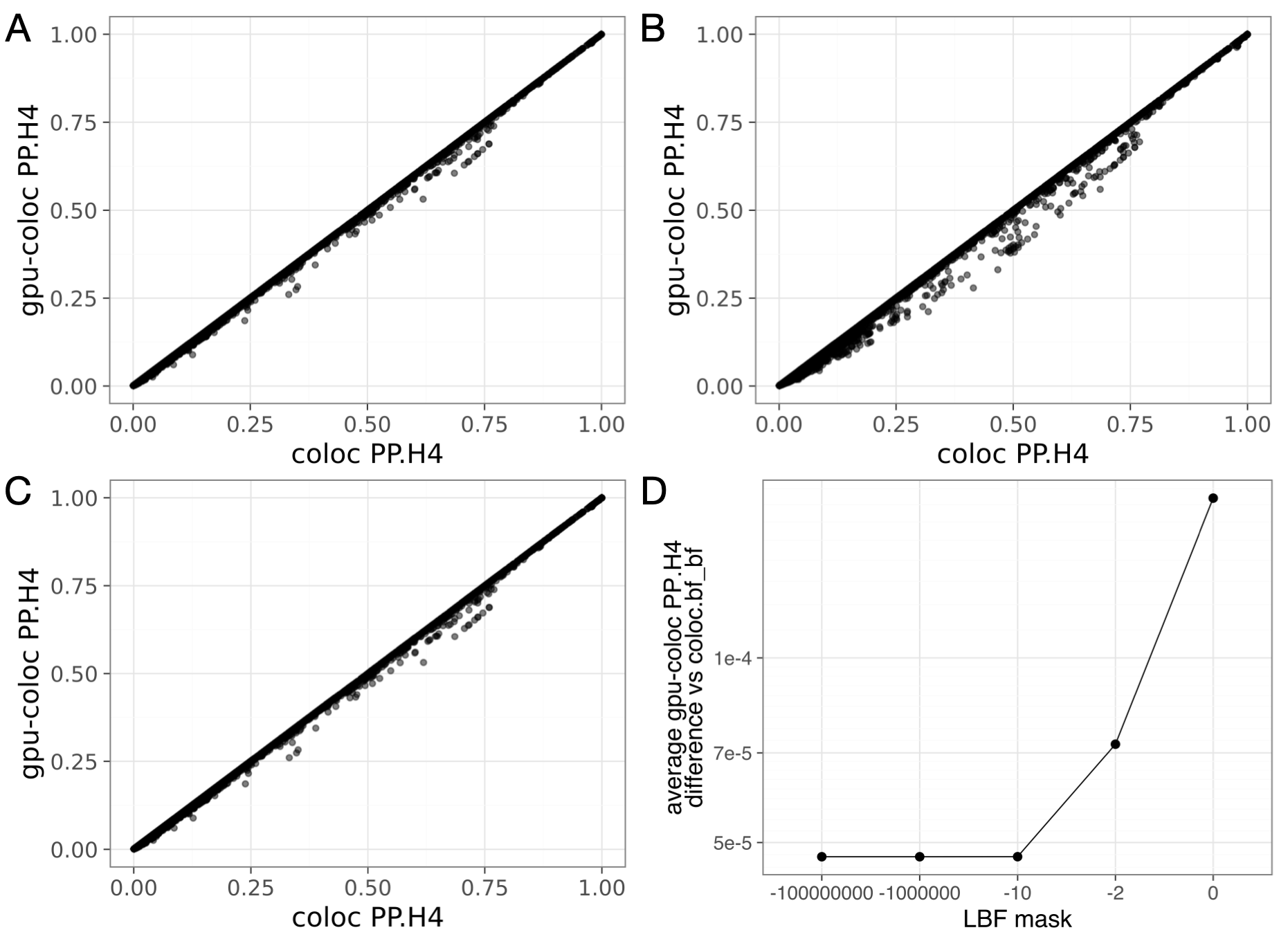

Supplement: S1 Fig — (A–C) Pairwise comparison of PP.H4 between gpu-coloc and coloc.bf_bf at LBF mask values of the gpu-coloc default −10⁶ (A), 0 (B), and −10 (C). (D) Mean PP.H4 difference between coloc.bf_bf and gpu-coloc as a function of LBF mask value (y-axis on log scale). (TIFF) [file pgen.1012209.s004.tiff]

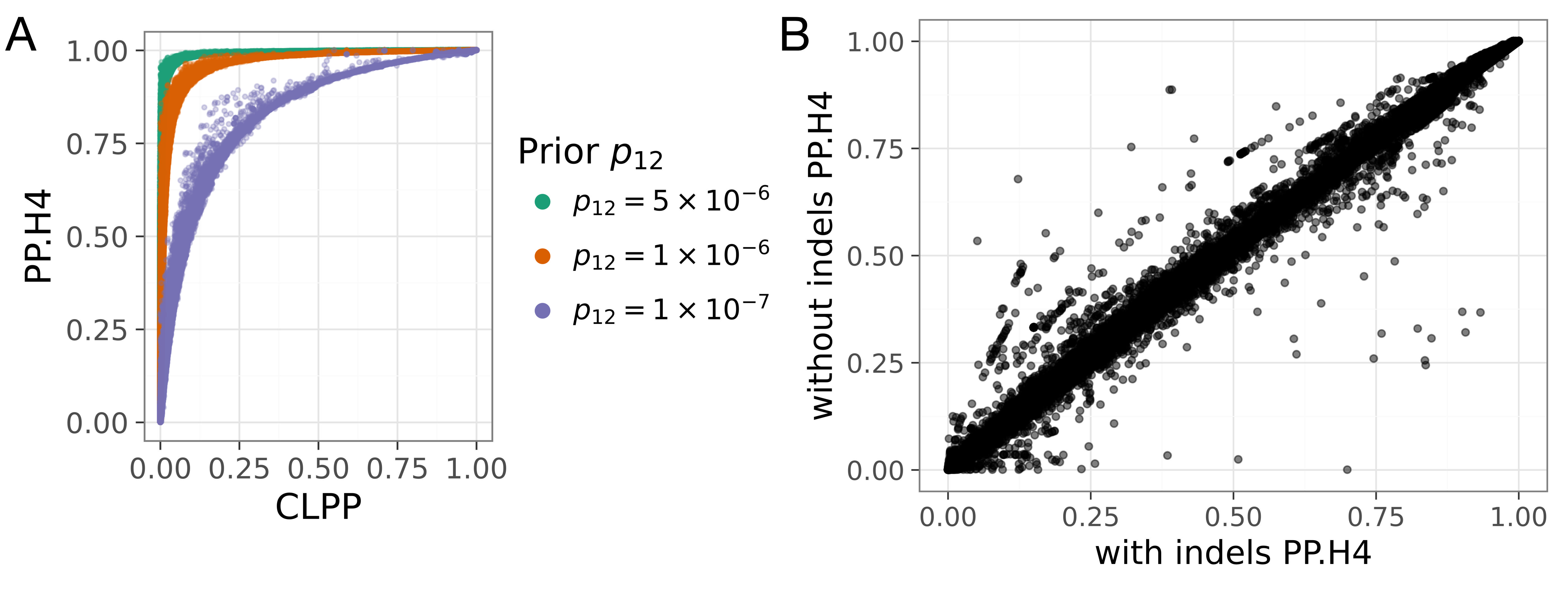

Supplement: S2 Fig — (A) Posterior probability comparison between gpu-coloc and CLPP across varying prior parameterisations (p12 = 5 × 10^−6^, 1 × 10^−6^, and 1 × 10^−7^) in the Rahu et al., 2025 metabolic trait GWAS versus eQTL Catalogue colocalisation. (B) Comparison of PP.H4 values while including or excluding all indel variants from the metabolic trait GWAS results. (TIFF) [file pgen.1012209.s005.tiff]

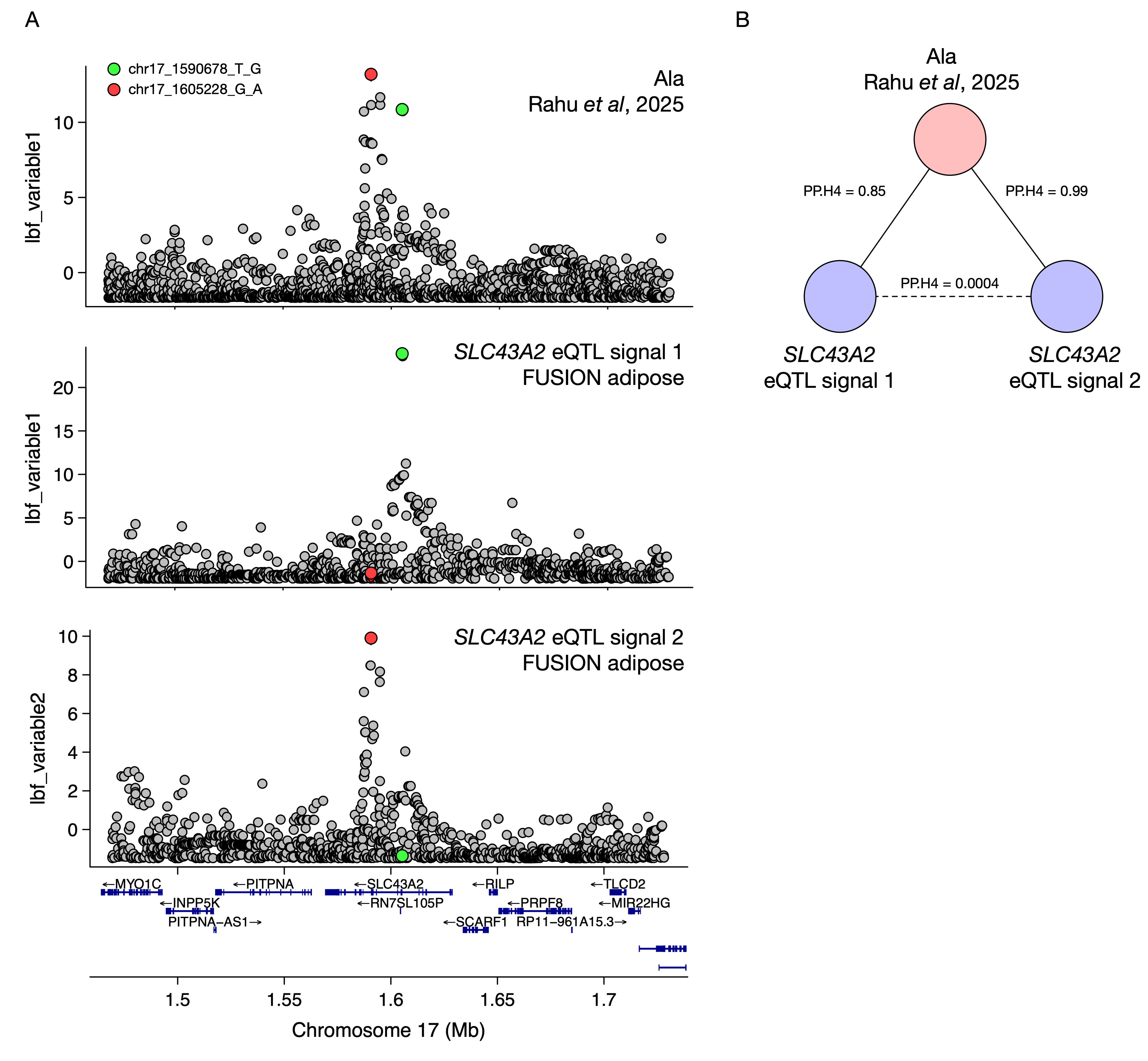

Supplement: S3 Fig — (A) Fine-mapped association signals for Alanine (Ala) and two independent eQTLs for SLC43A2 in the FUSION adipose eQTL dataset. The two lead eQTL variants (chr17_1590678_T_G and chr17_1605228_G_A) are in low LD with each other (r2 = 0.02). (B). Pairwise PP.H4 colocalisation posterior probabilities between the three association signals. The two eQTL signals both colocalise with the (unsuccessfully) fine mapped GWAS signal for Ala (PP.H4 = 0.85 and PP.H4 = 0.99, respectively), but they do not colocalise with each other (PP.H4 = 0.0004). (TIFF) [file pgen.1012209.s006.tiff]

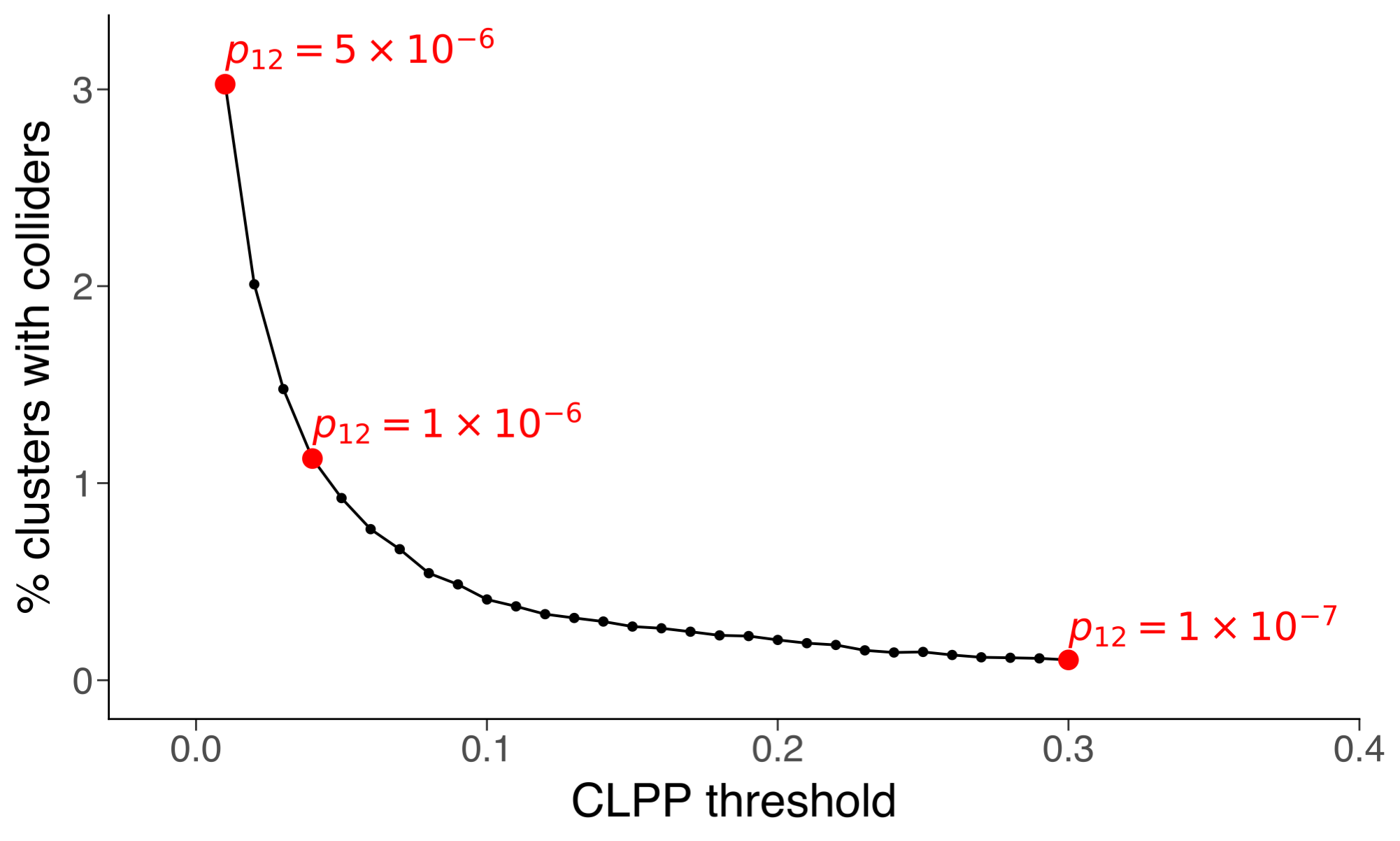

Supplement: S4 Fig — The red dots illustrate the CLPP thresholds and p12 prior probabilities at which CLPP and gpu-coloc (PP.H4 > 0.8) produce comparable results. (TIFF) [file pgen.1012209.s007.tiff]

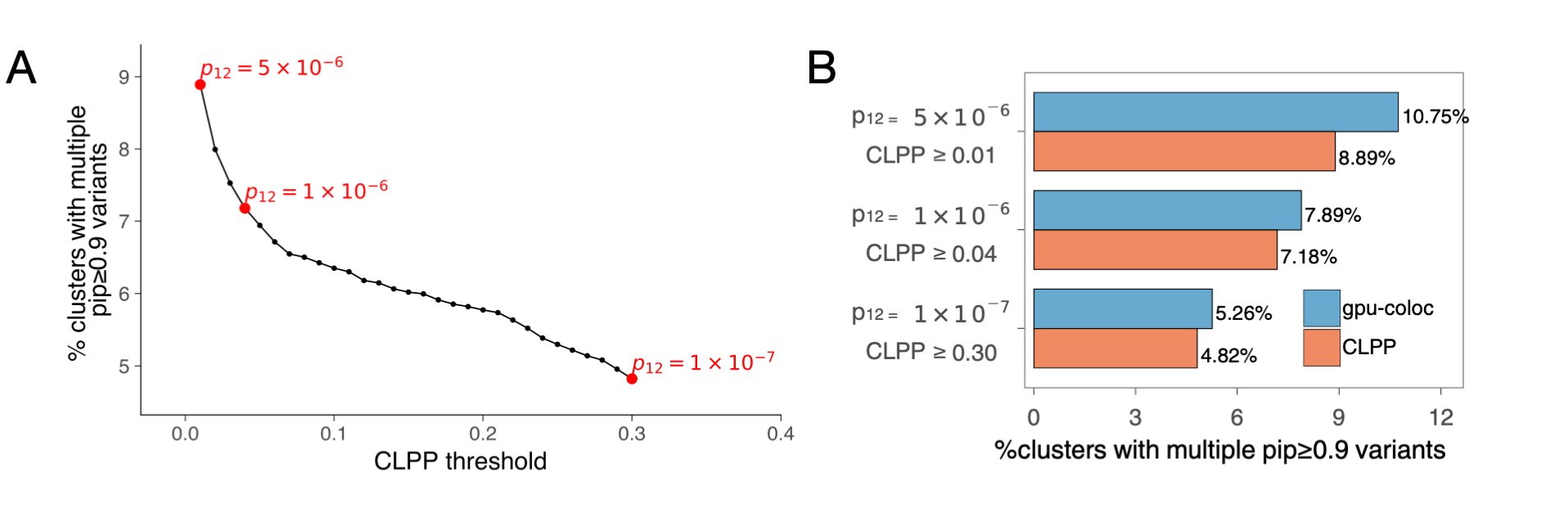

Supplement: S5 Fig — (A) Effect of the CLPP threshold on the percentage of clusters containing at least two distinct fine mapped variants (PIP ≥ 0.9). (B) Effect of p12 prior probability and CLPP threshold on the percentage of clusters containing at least two distinct fine mapped variants (PIP ≥ 0.9). For all p12 values, PP.H4 threshold was set to PP.H4 ≥ 0.8. (TIFF) [file pgen.1012209.s008.tiff]

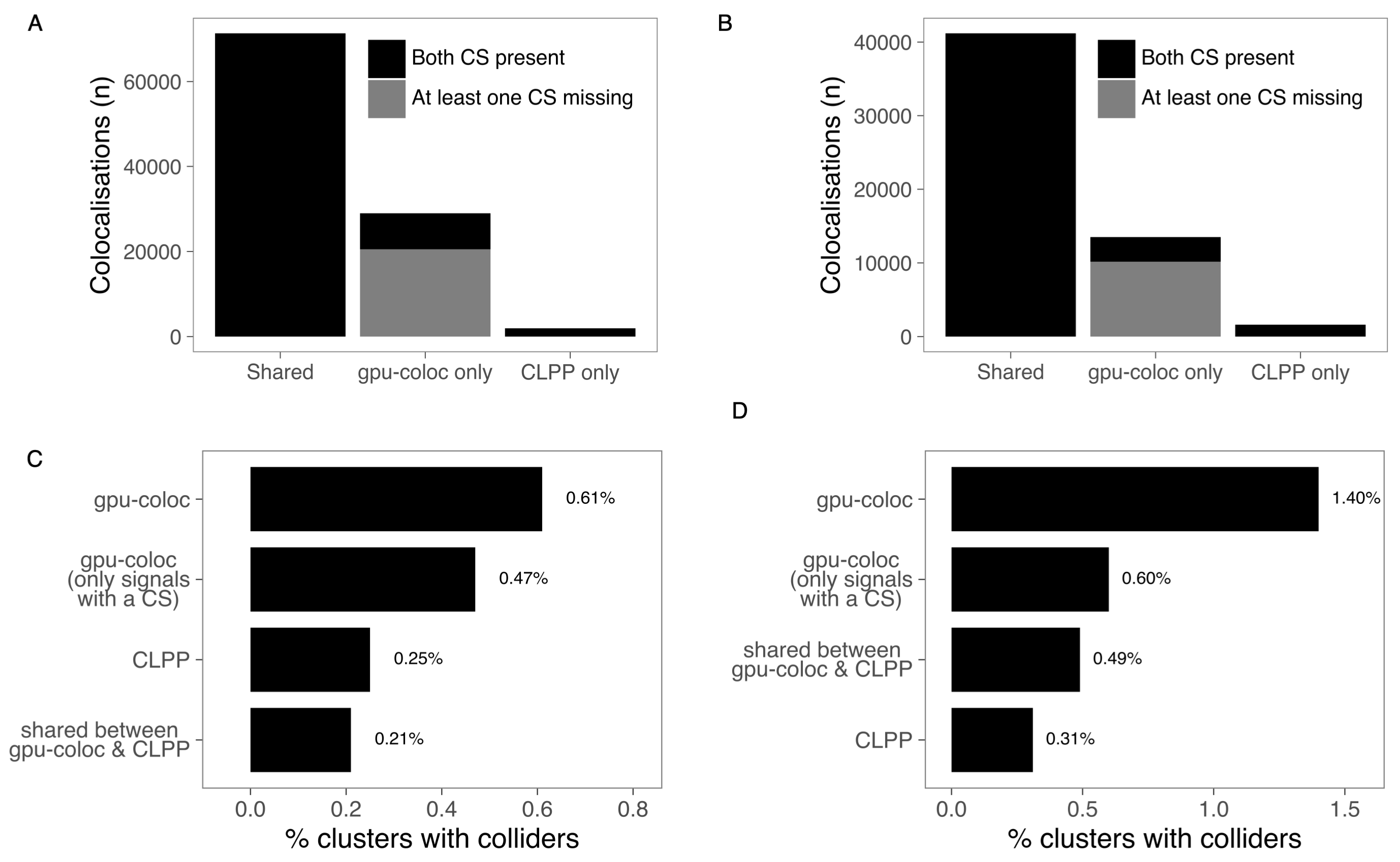

Supplement: S6 Fig — (A) Sources of difference from colocalisation between FinnGen versus eQTL Catalogue. (B) Sources of difference from colocalisation between 56 UK Biobank metabolic traits from Rahu et al., 2024 versus eQTL Catalogue. (C-D) Rate of colocalisation colliders in the eQTL Catalogue vs FinnGen r12 (Panel C) and eQTL Catalogue vs Rahu et al., 2025 (Panel D) analyses. Barplots show the percentage of clusters containing at least one colocalisation collider for all gpu-coloc colocalisation results (CLPP ≥ 0.04 and p12 = 1 × 10^−6^, PP.H4 ≥ 0.8), for gpu-coloc results where both signals have credible sets, for colocalisation results shared by gpu-coloc and CLPP, and all CLPP colocalisation results. (TIFF) [file pgen.1012209.s009.tiff]

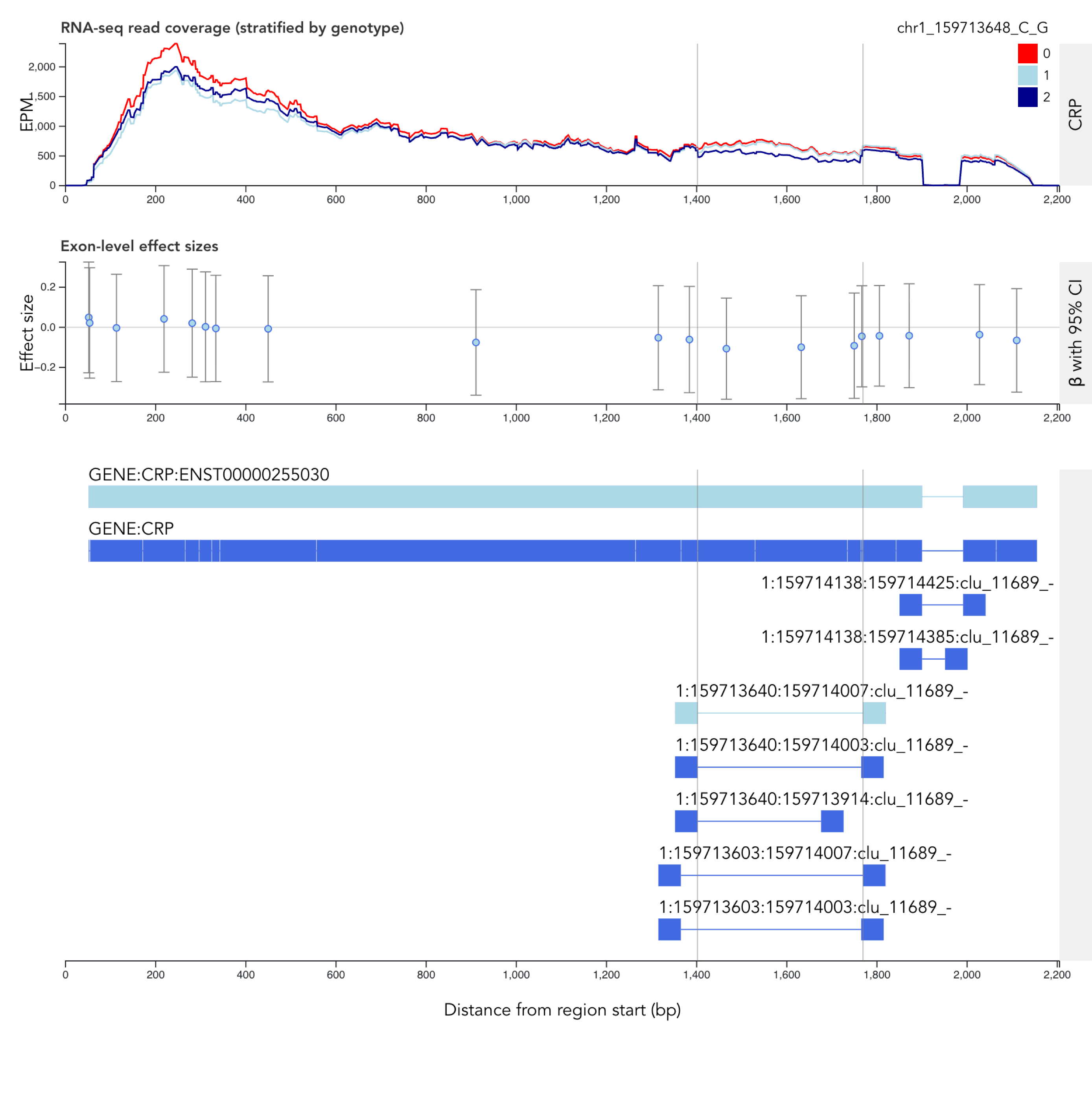

Supplement: S7 Fig — RNA-seq read coverage across the CRP gene in the GTEx liver dataset (QTD000270) is stratified by the genotype of the lead sQTL variant (rs1800947). The interactive plot can be viewed in the ELIXIR-Estonia eQTL Catalogue Browser (https://elixir.ut.ee/eqtl/?credible_set=QTD000270_1%3A159713640%3A159714007%3Aclu_11689_-_L1). (TIFF) [file pgen.1012209.s010.tiff]

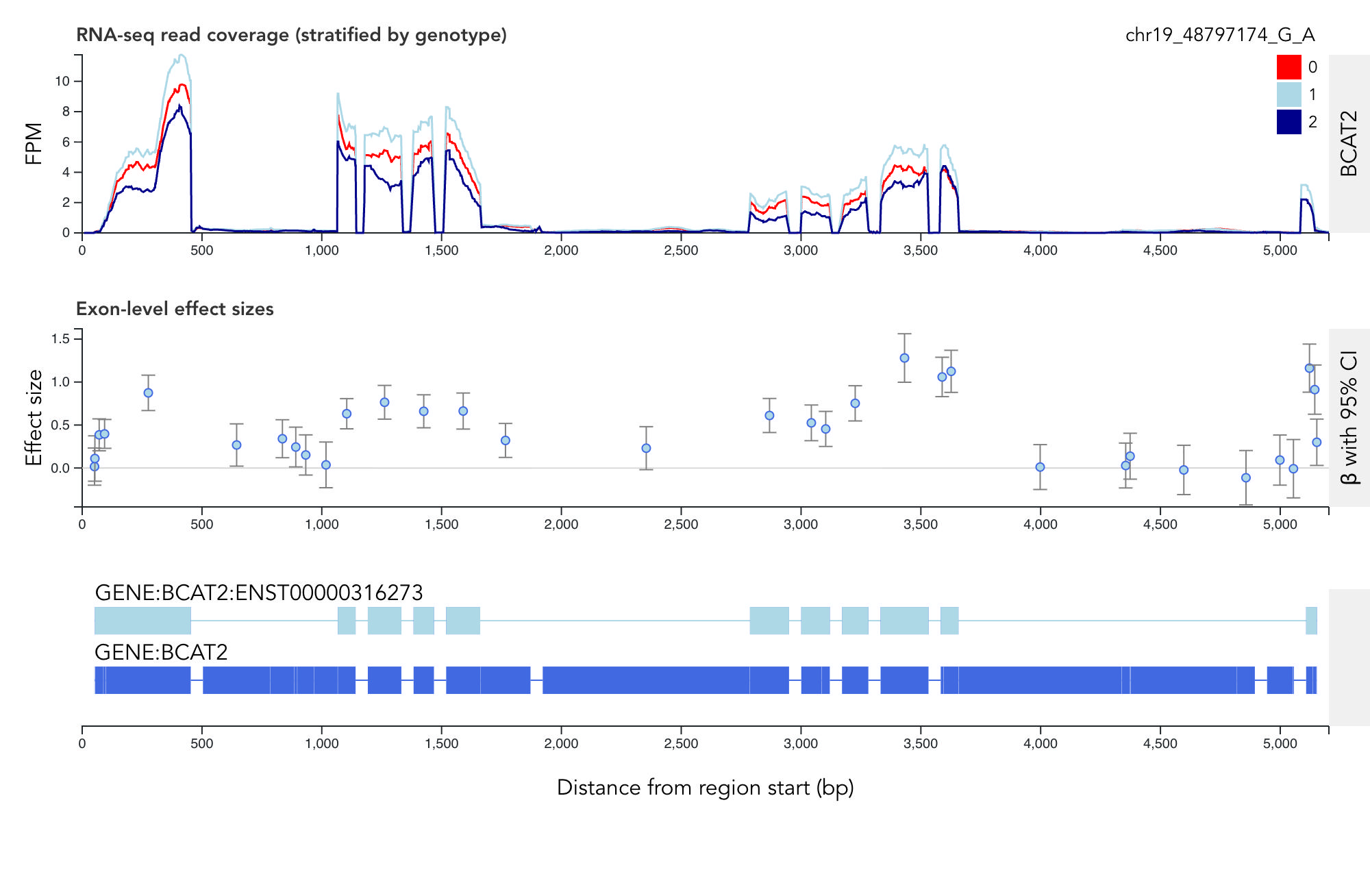

Supplement: S9 Fig — RNA-seq read coverage across the BCAT2 gene in the FUSION adipose tissue dataset (QTD000090) stratified by the genotype of the lead eQTL variant (rs35230038). The interactive plot can be viewed in the ELIXIR-Estonia eQTL Catalogue Browser (https://elixir.ut.ee/eqtl/?credible_set=QTD000090_ENSG00000105552_L1). (TIFF) [file pgen.1012209.s012.tiff]
