# Supplementary material for: Ultra-fast genetic colocalisation across millions of association signals
Source: PLoS Genet. 2026 Jun 17;22(6):e1012209. doi: 10.1371/journal.pgen.1012209 (PMC13289940; doi:10.1371/journal.pgen.1012209)
Supplement: Attachment — Submitted filename: Response to reviewers.pdf [file pgen.1012209.s014.pdf]

We thank the reviewers for the constructive and highly informative feedback. In addition to many minor edits, we have now made the following major revisions:

1. We have now significantly expanded the benchmarking of gpu-coloc, demonstrating that while the time spent per colocalisation test stayed flat or even increased for coloc.bf_bf (R implementation), gpu-coloc became relatively more efficient as the number of parallel tests increased (Figure 2B). Surprisingly, we also found that gpu-coloc maintained most of its performance gains even when run using a CPU backend, alleviating the need for expensive GPU hardware when running gpu-coloc.
2. We have added new analysis to assess the impact of missing genetic variants on colocalisation results (Figure 2C).
1. We have included new sensitivity analysis to assess how replacing log Bayes factors for missing genetic variants with various masking values affects colocalisation (Figure S1).

The page and line numbers in this document correspond to the “Revised Article with Changes Highlighted” file.

Reviewers' comments:

Reviewer's Responses to Questions**.**

Reviewer #1: The authors introduced gpu-coloc, a GPU-accelerated re-implementation of the coloc algorithm that enables biobank-scale colocalisation. Through comprehensive computational benchmarking, they demonstrated the efficiency of gpu-coloc. They also investigated the colocalisation colliders and provided practical recommendations in real data analysis for users. I have some comments as follows.

1. Although the authors explained PP.H4 in detail in the Methods section, it would be helpful to briefly introduce its meaning when it is first mentioned. This would make the manuscript more accessible to a more general audience.

We have now clarified this in the introduction (p2, lines 58-61):

“Moreover, while CLPP is defined at the variant level, coloc’s posterior probability that two association signals share a causal variant (PP.H4) is defined at the locus level, making it tricky to compare results from these two methods to each other.”

2. Line 153-156: Why is the gpu-coloc PP.H4 considered more conservative than that from coloc.bf_bf? According to Figure 2A, the PP.H4 values between the two methods are virtually identical, with only a few outliers where gpu-coloc reports lower values. Notably, these discrepancies are concentrated in the region where PP.H4 < 0.6, which may have limited impact on the false positive rate in practice. Furthermore, I don't think that being more conservative is necessarily superior.

We thank the Reviewer for this observation. Our intent was to highlight that PP.H4 values from gpu-coloc were very slightly lower than those from coloc.bf_bf across the comparison, but we agree that this difference is minor and noticeable in the PP.H4 range where its

practical impact is limited. We also agree that framing gpu-coloc as more conservative is not appropriate, as the methods are equivalent. We have therefore deemed the original sentence redundant and removed it.

3. Line 164-174: The major contribution of this paper lies in its highly efficient implementation. Therefore, I suggest providing more detailed information when benchmarking the computation time, such as the hardware of the high-performance computing system (GPU model, CPU model, number of cores, RAM), computation configuration (number of parallel processes), and the time spent on model fitting versus I/O operations. Additionally, how does the computation time of gpu-coloc scale with the dataset size? Identifying the primary computational bottleneck would be highly beneficial for potential users.

We thank the reviewer for this excellent suggestion. We have now significantly expanded our benchmarking which we now report in the updated section of the manuscript (pages 6-7, lines 222-247):

> *"We next compared the time spent per colocalisation test between gpu-coloc and coloc.bf_bf while varying the number of colocalisation tests from 230 (single GWAS vs a single eQTL dataset) to 359,698 (all GTEx eQTLs vs all metabolic trait associations) (Figure 2B). We ran all benchmarks single-threaded and removed all signals with maximum LBF < 5, as we have found these do not result in colocalisations. We tested gpu-coloc with all three Torch backends: CUDA GPU, Mac Metal GPU, and CPU. We found that while the time spent per colocalisation test stayed flat or even increased for coloc.bf_bf, gpu-coloc became more efficient as the number of parallel tests increased (Figure 2B). When benchmarking just the colocalisation step of the analysis on the University of Tartu HPC system (thus excluding data input-output (IO) and various pre-processing steps), the gpu-coloc with CUDA GPU backend was 795-fold faster compared to the R implementation and the gpu-coloc with the CPU backend was 190-fold faster on the same node (see Methods). Total speed-ups (including IO and pre-processing steps) of the two backends were 1658-fold and 516-fold, respectively (Table S2). As the GPU backends speedup cannot be disentangled from the more powerful hardware (see Methods), we also benchmarked coloc.bf_bf with our gpu-coloc on an M2 Max 14" MacBook Pro with 32 GB RAM. Compared to R, the gpu-coloc with Metal GPU backend yielded a 369-fold speedup in the colocalisation step and, surprisingly, the CPU backend a 416-fold speedup in the colocalisation step, with a total speedup of 1,170-fold (Table S3). This demonstrates that gpu-coloc can also be successfully run on machines without GPUs, while still maintaining the large performance gains over a naive R implementation."*

For your convenience, here is also the updated version of Figure 2 with panels B and C added:

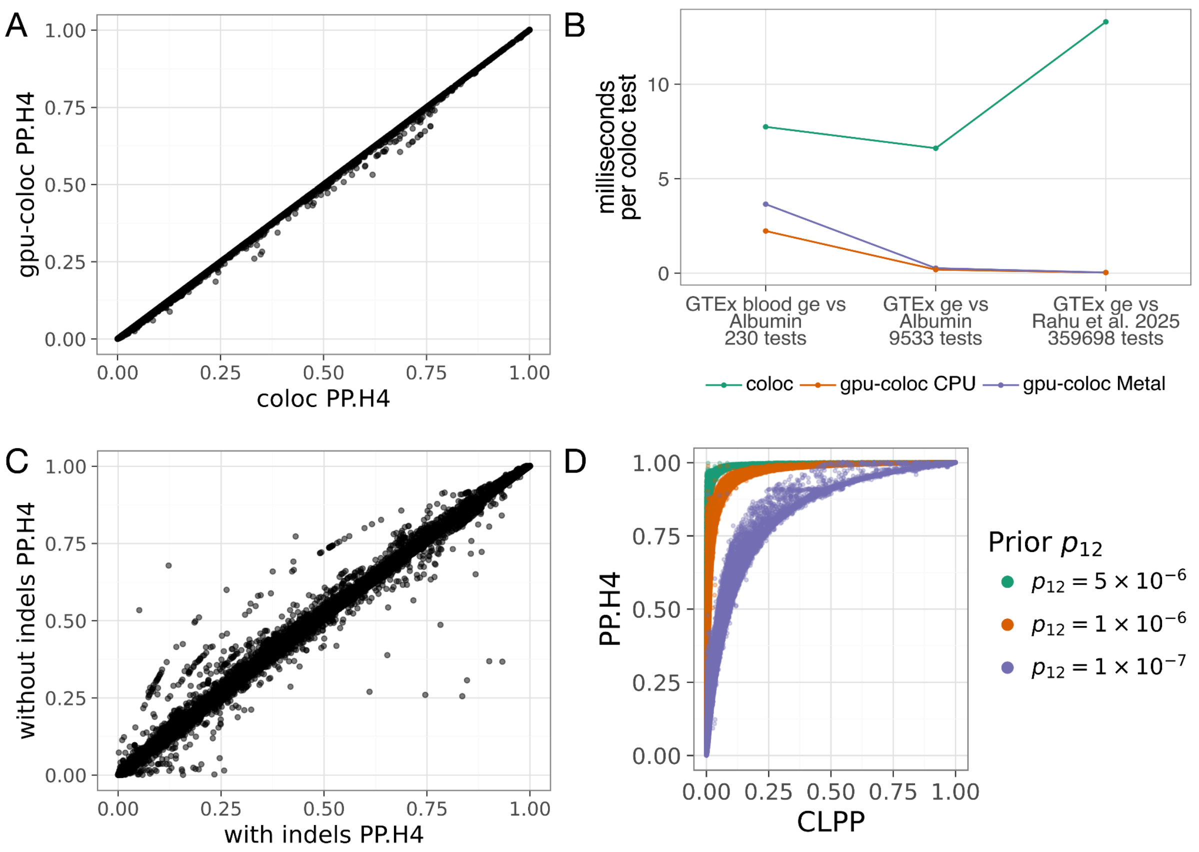

Figure 2. Comparison of the gpu-coloc, coloc.bf_bf, and CLPP colocalisation methods. (**A**) Comparison of the PP.H4 values between gpu-coloc and coloc.bf_bf. (**B**) Time spent in milliseconds per colocalisation test between coloc.bf_bf, and gpu-coloc with CPU and Metal backends. (**C**) Comparison of PP.H4 values while including and excluding indels. (**D**) Posterior probability comparison between gpu-coloc and CLPP across varying prior parameterisations (p_{12} = 5×10^{-6}, 1×10^{-6}, and 1×10^{-7}) in the FinnGen *versus* eQTL Catalogue colocalisation.

We have also added more details about the benchmarks to the Methods section:

> *"To benchmark the performance of gpu-coloc, we compared it with the original coloc.bf_bf implementation in R. Both implementations were applied to chromosome 19 in three batches of increasing size between the GTEx[22] datasets included in the eQTL Catalogue and the Rahu et al., 2025[23] metabolic traits GWAS signals:*
>
> 1. *GTEx blood gene expression (560 fine mapped signals) against the Albumin GWAS (19 signals);*
> 2. *All GTEx gene expression datasets (17,598 signals) against the Albumin GWAS;*
> 3. *All GTEx gene expression datasets against all metabolic traits GWAS from Rahu et al., 2025[23] (1,127 signals).*
>
> *We used prior probabilities $p_1 = p_2 = 1\times10^{-4}$ and $p_{12} = 1\times10^{-6}$ throughout. Benchmarks were performed in three hardware configurations, all on a single thread. The R implementation of coloc and the gpu-coloc CPU backend were run on an Ares CPU*

node of the University of Tartu HPC cluster (AMD EPYC 7702 64-core processor, 1 core and 30 GB RAM allocated, 8 TB SSD, HDR InfiniBand at 100 Gbps). The gpu-coloc GPU backend was run on a Firefly GPU node of the same cluster (AMD EPYC 9575F 64-core processor, 1 core and 30 GB RAM allocated, 1 NVIDIA H200 GPU with 141 GB VRAM, HDR InfiniBand at 100 Gbps). For a same-hardware comparison, we also ran the R implementation, the gpu-coloc CPU backend, and the gpu-coloc Metal GPU backend on a MacBook Pro with an Apple M2 Max and 32 GB RAM."

4. How does the computational efficiency of gpu-coloc compare with other popular efficient methods (e.g., HyPrColoc, https://doi.org/10.1038/s41467-020-20885-8)? I suggest including more competing methods for a more comprehensive comparison.

We note that HyPrColoc does not perform the same test as coloc: rather than evaluating all pairwise colocalisations, it computes the posterior probability of full colocalisation across n traits, approximated as the product of 1) the probability that all traits share an associated region and 2) the probability that these shared associations align at a single causal variant. Because HyPrColoc and coloc answer different statistical questions, a direct comparison of their computational resources would be misleading. However, we have now included Torch CPU backend into our benchmarks, and found that gpu-coloc maintains most of its performance advantages even if GPUs are not available (see response to point 3 above).

5. The empirical strategy to select the p12 prior and corresponding CLPP thresholds by quantifying the rate of colocalisation colliders is very interesting and valuable to users. However, relying solely on this metric may be limiting since the colocalisation colliders are not strictly equivalent to false positives. Have the authors considered other metrics?

We thank the reviewer for this comment. We agree that colocalisation colliders are not strictly equivalent to false positives and we have softened the wording accordingly on p9, 358-359:

"While these colocalisation colliders are technically not false positives (there is still at least one shared causal variant in both studies, see example on Figure S3)."

We have now also performed an additional analysis using an alternative definition of colocalisation colliders, which we report on page 9, lines 380-385:

"As an alternative strategy to detect colocalisation colliders, we quantified the number of colocalisation clusters that contained credible sets with two or more confidently fine mapped genetic variants (PIP > 0.9). We observed a broadly similar trend where the proportion of clusters containing at least one collider event decreased at more stringent colocalisation thresholds (Figure S5). However, the overall collider rate was higher with this metric, perhaps because variant-level PIPs might not always be

perfectly calibrated.[31]"

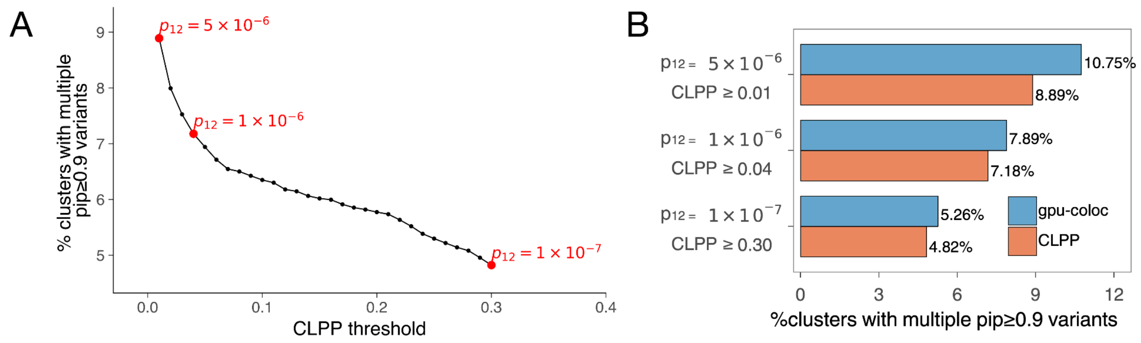

Figure S5. Effect of CLPP threshold and gpu-coloc priors on the percentage of clusters containing at least two credible sets with distinct fine mapped variants (PIP ≥ 0.9). (**A**) Effect of the CLPP threshold on the percentage of clusters containing at least two distinct fine mapped variants (PIP ≥ 0.9). (**B**) Effect of p_{12} prior probability and CLPP threshold on the percentage of clusters containing at least two distinct fine mapped variants (PIP ≥ 0.9). For all p_{12} values, PP.H4 threshold was set to PP.H4 ≥ 0.8.

6. It's interesting to see that the SuSiE signals that failed purity filtering may cause the increased rate of colocalisation colliders for gpu-coloc. In the real data analyses, did the authors observe any concrete examples of such collider events? For example, do these signals exhibit broader or multi-modal PIP distributions, or more complex LD structures that prevent them from passing the purity filtering?

We now illustrate one such real collider event on Figure S3. In this case, it does not seem that the collider is caused by a particularly complex LD pattern, but rather by limited statistical power to distinguish between the two independent causal variants in GWAS fine mapping:

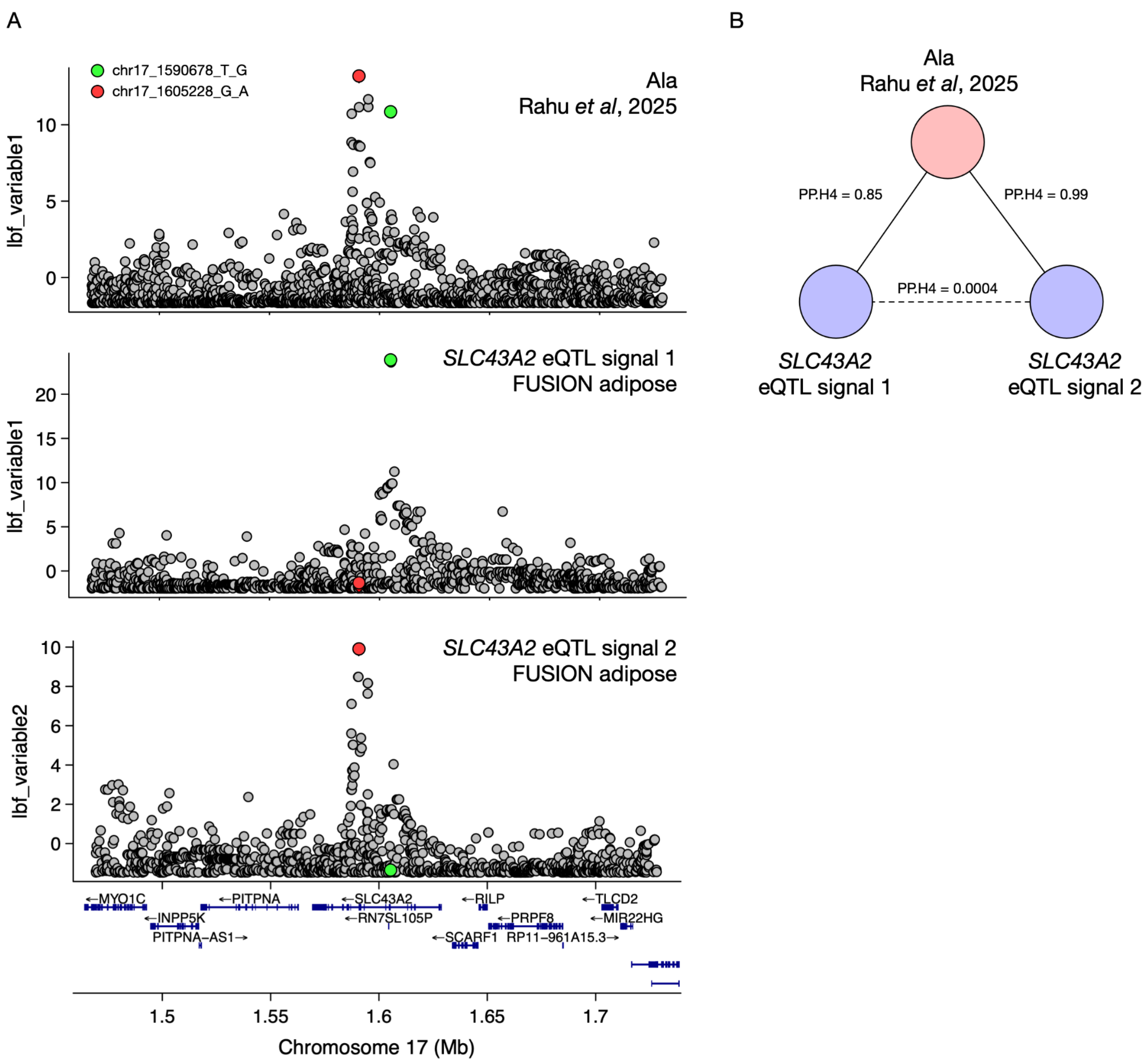

Figure S3. Example of a colocalisation collider identified in the eQTL Catalogue and metabolic trait GWAS colocalisation analysis. (A) Fine-mapped association signals for Alanine (Ala) and two independent eQTLs for SLC43A2 in the FUSION adipose eQTL dataset. The two lead eQTL variants (chr17_1590678_T_G and chr17_1605228_G_A) are in low LD with each other (r^2 = 0.02). (B). Pairwise PP.H4 colocalisation posterior probabilities between the three association signals. The two eQTL signals both colocalise with the (unsuccessfully) fine mapped GWAS signal for Ala (PP.H4 = 0.85 and PP.H4 = 0.99, respectively), but they do not colocalise with each other (PP.H4 = 0.0004).

While we agree that it would be interesting to explore in greater detail, what are the root causes of colocalisation colliders, we believe that this analysis is out of the scope of the current paper.

7. Figure 3C: Does CS refer to credible set? I suggest explaining the abbreviation at first use.

We thank the reviewer for bringing this to our attention. We have added the abbreviation to the caption of Figure 3.

Reviewer #2: In this manuscript, the authors introduced a scalable implementation of colocalization algorithm. They compared locus-level posteriors with variant-level overlap metrics such as CLPP and provided practical recommendations for choosing pipelines, thresholds, and priors in colocalization analyses. The work is likely to be impactful for the statistical genetics community, particularly for large-scale integrative analyses involving GWAS and molecular QTL datasets. However, several aspects of the study would benefit from clearer explanation, including implementation choices for handling missing values, the design of benchmarking, and parts of the practical recommendations. Detailed comments are provided below:

1. In the title, "across millions of traits" is potentially misleading. As presented in the following analyses, it appear to involve millions of colocalization tests or locus-trait or signal-trait pairs, rather than millions of distinct traits. Rephrasing the title to more precisely reflect the scale of the analyses would improve clarity.

We agree that the original title could have been misleading, we have now changed the title to “Ultra-fast genetic colocalisation across millions of **association signals**”.

2. The workaround of replacing missing LBF values with an extreme negative constant is a critical implementation step that enables the matrix-based computations in gpu-coloc. To improve transparency, It would be helpful to explicitly illustrate this step in Figure 1. In addition, please justify the choice of the value of -1e6. It is unclear whether this threshold should remain fixed across different datasets or analysis settings, or whether it should depend on factors such as sample size, effect size distributions, or prior choices. A brief sensitivity analysis assessing the robustness of posterior inferences to the choice of this constant would further strengthen confidence in the method.

This is indeed a critical step and the value was chosen by empirically testing 0, −2, −10, -10^6, and -10^8. For values ≥ −10 we observed roughly twice as many spurious pairs unique to gpu-coloc, both relative to the R implementation and relative to gpu-coloc runs with smaller replacements. These spurious pairs passed our default posterior threshold despite being driven by masked variants. From -10^6 onwards the result stabilised, so we recommend -10^6 as a safe default. We note that our approach is related to the "masking" strategy proposed in https://doi.org/10.1371/journal.pgen.1008720, which uses a value of 0, assuming no correlation, but allowing masked variants to contribute spurious support to other configurations.

We now explicitly note the masking step on Figure 1:

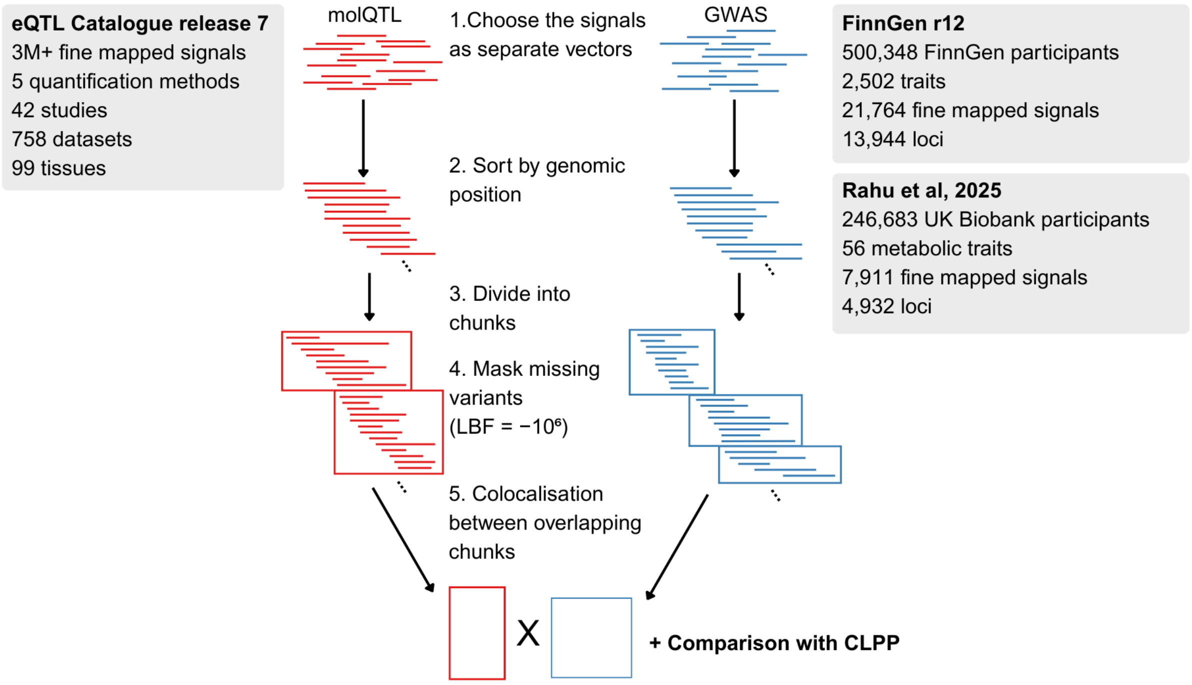

We also discuss the sensitivity analysis on page 4, rows 155-158:

> *"This mask was chosen via sensitivity analysis across values of 0, -2, -10, -10^6, and -10^8. The mean difference from coloc.bf_bf plateaued at masks ≤ -10, but -10^6 was preferred over -10 as it did not induce artefact results (Figure S1)."*

And to support this, we have also added a new supplementary figure (Figure S1):

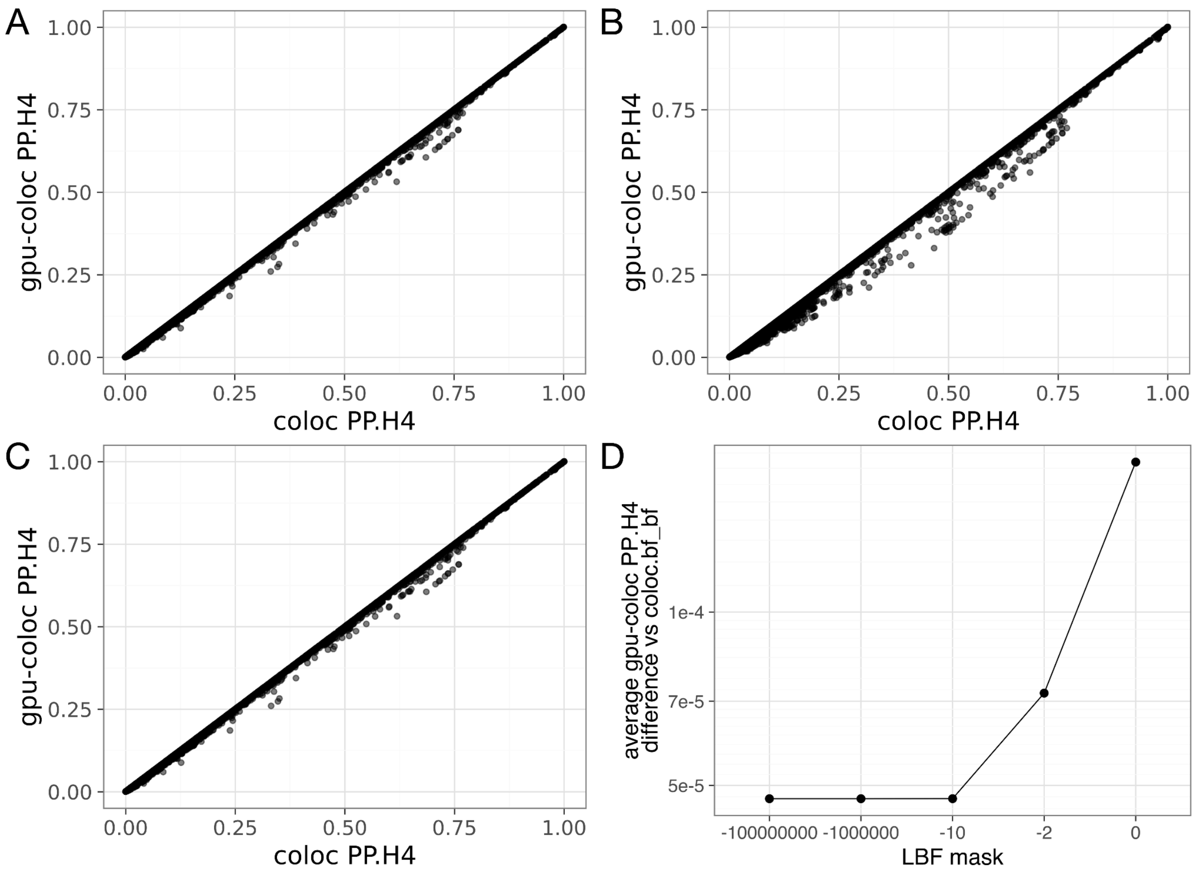

Figure S1. Sensitivity of gpu-coloc PP.H4 to the LBF mask value. (**A–C**) Pairwise comparison of PP.H4 between gpu-coloc and coloc.bf_bf at LBF mask values of the gpu-coloc default -10^6 (**A**), 0 (**B**), and −10 (**C**). (**D**) Mean PP.H4 difference between coloc.bf_bf and gpu-coloc as a function of LBF mask value (y-axis on log scale).

3. The gpu-coloc replaces missing LBFs with an extreme negative value, so the difference in PP.H4 shown in Figure 2A may depend on the pattern and extent of missingness across datasets, traits, locus, and variants. Please clarify how frequently the replacement occurs in practice, and whether heterogeneity in missingness could influence the observed differences between methods.

We note that the expected level of missingness will highly depend on the type and source of summary statistics included in the analysis. For example, the FinnGen and Rahu et al, 2025 datasets tested the exact same set of variants for all traits and thus contain no missing values. However, we have now performed additional analysis to quantify the impact of missing values on colocalisation results, which we report on pages 7-8, rows 264-303:

Effect of missingness on colocalisation between datasets

As demonstrated above (Figure 2B), gpu-coloc achieves the largest speed-up when performing many-to-many colocalisation tests. However, an important limitation of this approach is that if some association signals contain missing genetic variants, then these need to be masked globally (with coloc.bf_bf, missing variants are handled separately for each pairwise colocalisation test). To quantify the impact of

masking missing values, we performed colocalisation between GTEx eQTLs and Rahu et al., 2025 metabolic trait signals twice: once with indels included for all metabolic traits, and once after indels were removed from every other metabolic trait (so that they would be treated as missing variants and masked). We then restricted both analyses to the metabolic traits where indels had been excluded in one version of the analysis and compared the resulting colocalisation PP.H4 values to each other (Figure 2C). Of the 2,923 colocalising pairs (PP.H4 ≥ 0.8) detected when indels were included throughout, 113 (3.9%) no longer passed the trim_posterior step after indels were removed, and a further 18 (0.6%) fell below the PP.H4 ≥ 0.8 threshold. Conversely, of the 2,968 pairs detected in the analysis with indels removed, 150 (5.1%) had been below the PP.H4 threshold in the original analysis and 26 had not passed trim_posterior, suggesting these are false positives induced by masking of missing values (Figure 2C). Interestingly, the impact on colocalisation was very similar when indels were removed from all metabolic trait signals (and thus completely excluded from colocalisation testing) (Figure S2), suggesting that missingness itself has a larger impact on colocalisation than masking.

For you convenience, here is the relevant panel C from Figure 2:

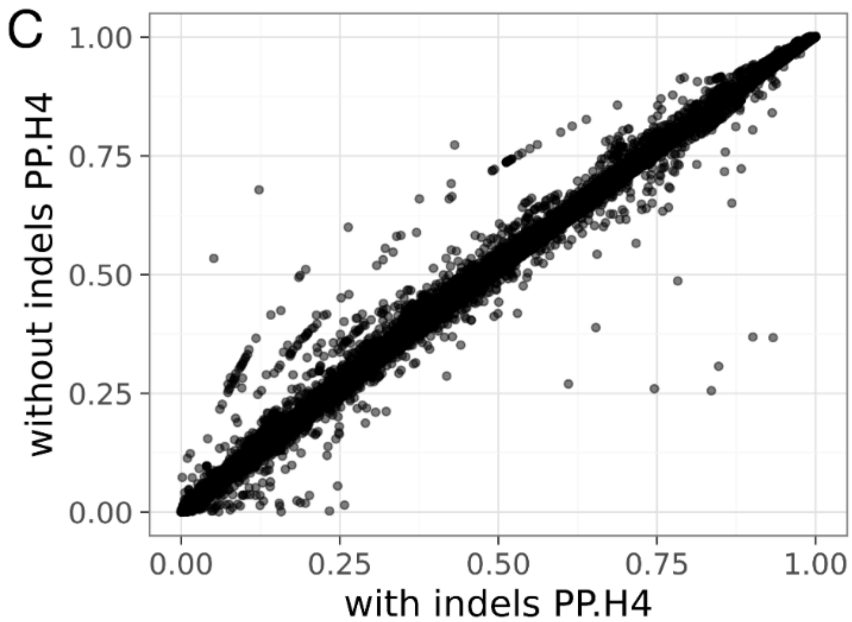

Figure 2. Comparison of the gpu-coloc, coloc.bf_bf, and CLPP colocalisation methods. (**C**) Comparison of PP.H4 values while including or excluding indels.

And the same plot from Figure S2 where indels have been completely excluded from gpu-coloc (without masking):

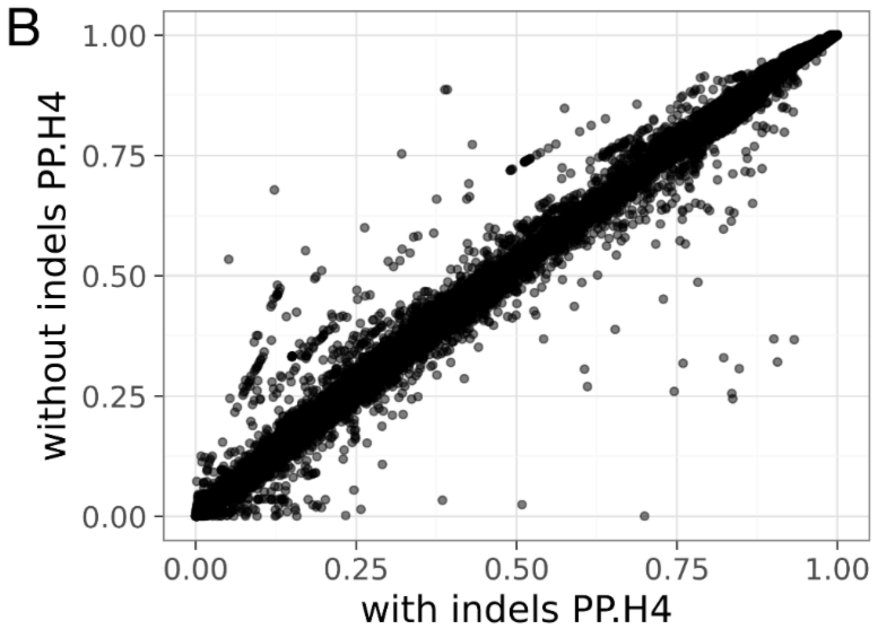

Figure S2. (**B**) Comparison of PP.H4 values while including or excluding all indel variants from the metabolic trait GWAS results.

These results indicate that the impact of missingness on colocalisation results is primarily driven by missingness itself (thus similarly affecting both coloc.bf_bf and gpu-coloc) rather than the particular masking strategy chosen.

4. The results of computational performance on pages 5-6 would benefit from additional clarification and reorganization. (1) When reporting the running times, it would be helpful to clearly specify the computational settings, including allocated memory, number of cores, and the total number of colocalization tests performed. Reporting a normalized metric such as running time per test may facilitate more meaningful comparison across methods and computing environments. (2) The manuscript mentions running times on a personal Mac computer in both lines 168 and 179, which appear to convey similar messages regarding computational efficiency. Reorganizing these descriptions could improve clarity and avoid redundancy. (3) Finally, to better demonstrate scalability, it would be informative to show how running time scales with the number of colocalization tests, and to compare with existing methods.

Reviewer 1 made a very similar suggestion (see point 3 above). As a result, we have now significantly expanded our benchmarking which we now report in the updated section of the manuscript (pages 6-7, lines 222-247):

> *“We next compared the time spent per colocalisation test between gpu-coloc and coloc.bf_bf while varying the number of colocalisation tests from 230 (single GWAS vs a single eQTL dataset) to 359,698 (all GTEx eQTLs vs all metabolic trait associations) (Figure 2B). We ran all benchmarks single-threaded and removed all*

signals with maximum LBF < 5, as we have found these do not result in colocalisations. We tested gpu-coloc with all three Torch backends: CUDA GPU, Mac Metal GPU, and CPU. We found that while the time spent per colocalisation test stayed flat or even increased for coloc.bf_bf, gpu-coloc became more efficient as the number of parallel tests increased (Figure 2B). When benchmarking just the colocalisation step of the analysis on the University of Tartu HPC system (thus excluding data input-output (IO) and various pre-processing steps), the gpu-coloc with CUDA GPU backend was 795-fold faster compared to the R implementation and the gpu-coloc with the CPU backend was 190-fold faster on the same node (see Methods). Total speed-ups (including IO and pre-processing steps) of the two backends were 1658-fold and 516-fold, respectively (Table S2). As the GPU backends speedup cannot be disentangled from the more powerful hardware (see Methods), we also benchmarked coloc.bf_bf with our gpu-coloc on an M2 Max 14" MacBook Pro with 32 GB RAM. Compared to R, the gpu-coloc with Metal GPU backend yielded a 369-fold speedup in the colocalisation step and, surprisingly, the CPU backend a 416-fold speedup in the colocalisation step, with a total speedup of 1,170-fold (Table S3). This demonstrates that gpu-coloc can also be successfully run on machines without GPUs, while still maintaining the large performance gains over a naive R implementation."

For your convenience, here is also the updated version of Figure 2 with panels B and C added:

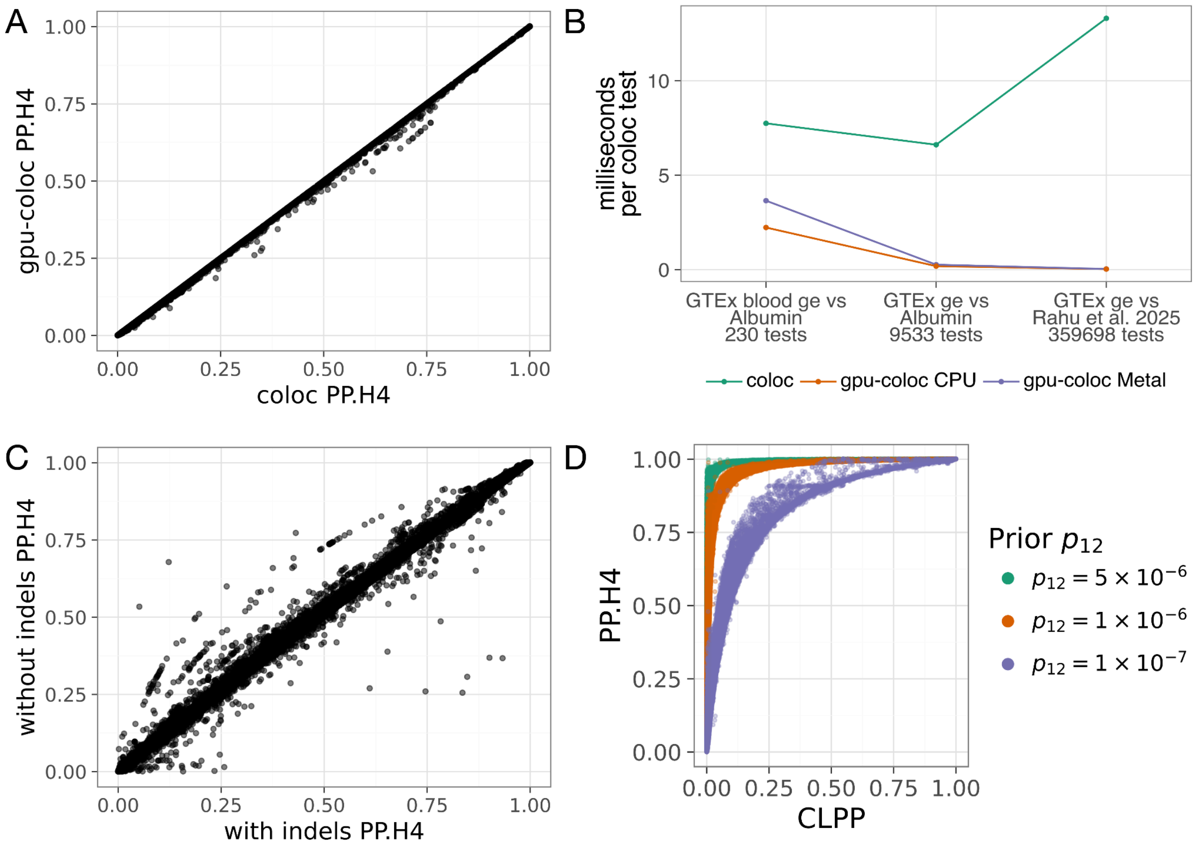

Figure 2. Comparison of the gpu-coloc, coloc.bf_bf, and CLPP colocalisation methods. (**A**) Comparison of the PP.H4 values between gpu-coloc and coloc.bf_bf. (**B**) Time

spent in milliseconds per colocalisation test between coloc.bf_bf, and gpu-coloc with CPU and Metal backends. (**C**) Comparison of PP.H4 values while including and excluding indels. (**D**) Posterior probability comparison between gpu-coloc and CLPP across varying prior parameterisations (p_{12} = 5×10⁻⁶, 1×10⁻⁶, and 1×10⁻⁷) in the FinnGen *versus* eQTL Catalogue colocalisation.

We have also added more details about the benchmarks to the Methods section:

> *"To benchmark the performance of gpu-coloc, we compared it with the original coloc.bf_bf implementation in R. Both implementations were applied to chromosome 19 in three batches of increasing size between the GTEx[22] datasets included in the eQTL Catalogue and the Rahu et al., 2025[23] metabolic traits GWAS signals:*
>
> 4. *GTEx blood gene expression (560 fine mapped signals) against the Albumin GWAS (19 signals);*
> 5. *All GTEx gene expression datasets (17,598 signals) against the Albumin GWAS;*
> 6. *All GTEx gene expression datasets against all metabolic traits GWAS from Rahu et al., 2025[23] (1,127 signals).*
>
> *We used prior probabilities $p_1 = p_2 = 1\times10^{-4}$ and $p_{12} = 1\times10^{-6}$ throughout. Benchmarks were performed in three hardware configurations, all on a single thread. The R implementation of coloc and the gpu-coloc CPU backend were run on an Ares CPU node of the University of Tartu HPC cluster (AMD EPYC 7702 64-core processor, 1 core and 30 GB RAM allocated, 8 TB SSD, HDR InfiniBand at 100 Gbps). The gpu-coloc GPU backend was run on a Firefly GPU node of the same cluster (AMD EPYC 9575F 64-core processor, 1 core and 30 GB RAM allocated, 1 NVIDIA H200 GPU with 141 GB VRAM, HDR InfiniBand at 100 Gbps). For a same-hardware comparison, we also ran the R implementation, the gpu-coloc CPU backend, and the gpu-coloc Metal GPU backend on a MacBook Pro with an Apple M2 Max and 32 GB RAM."*

5. The gpu-coloc is compared with coloc.bf_bf and with CLPP using different datasets. It would be helpful for the authors to clarify the rationale and criterion in choosing different datasets for different comparisons. In particular, please explain whether this choice reflects method-specific input requirements, data availability, or distinct benchmarking goals. Without such clarification, it is difficult to disentangle method differences from dataset-specific effects.

We thank the reviewer for highlighting this discrepancy. This distinction was arbitrary. The comparison between coloc.bf_bf and gpu-coloc on Figure 2A was restricted to GTEx eQTLs and Rahu et al, 2025 metabolic trait association signals from chromosome 19 due to computational reasons (running coloc.bf_bf on a larger dataset would have been prohibitively slow).

The comparison between gpu-coloc and CLPP was initially done using the eQTL Catalogue vs FinnGen colocalisation (Figure 2D). However, we have now repeated the gpu-coloc and CLPP comparison on the eQTL Catalogue vs Rahu et al, 2025 metabolic trait associations

(Figure S2A) and found highly concordant results leading to the same decision thresholds (i.e. we still recommend CLPP > 0.04 as a useful default threshold). This new analysis is now also mentioned in the main text (page 8, lines 325-326):

> "Colocalisation between Rahu *et al.* 2025 metabolic traits and eQTL Catalogue revealed similar results (Figure S2A)."

For convenience, here is the newly added Figure S2A:

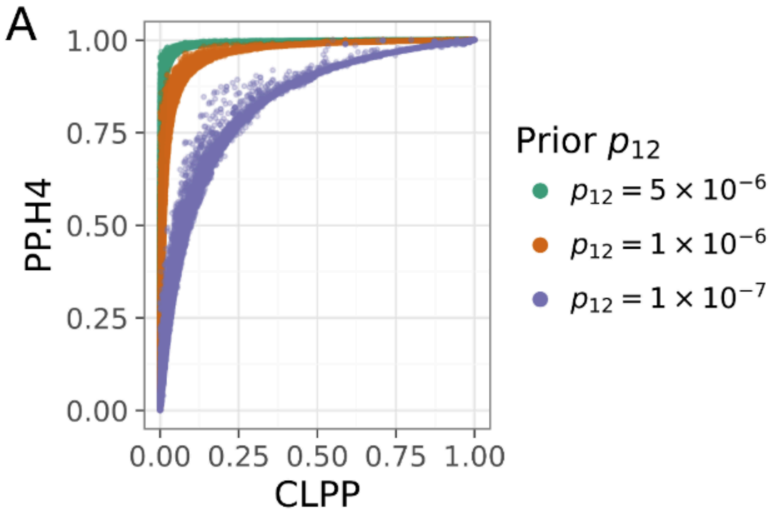

Figure S2. (A) Posterior probability comparison between gpu-coloc and CLPP across varying prior parameterisations (p_{12} = 5×10^{-6}, 1×10^{-6}, and 1×10^{-7}) in the Rahu *et al.*, 2025 metabolic trait GWAS *versus* eQTL Catalogue colocalisation.

7. From "we leveraged our efficient gpu-coloc implementation to calculate both the CLPP and PP.H4 values", it seems the gpu-coloc method can be used as re-implementation for both standard coloc and CLPP. But in the method overview and Figure 1, it seems gpu-coloc is only a more efficient tool for standard coloc. Please clarify.

We thank the reviewer for bringing attention to this discrepancy. The calculations were independent, PP.H4 was calculated using gpu-coloc and CLPP using the CLPP method. We have clarified this now on page 8, lines 314-316:

> *"To explore this question in more detail, we calculated both the CLPP and gpu-coloc PP.H4 values for approximately 200 million molQTL and GWAS signal pairs across the eQTL Catalogue and FinnGen datasets."*

8. The mapping between CLPP and coloc posterior probability (page 6-7) is derived empirically using the eQTL Catalogue and FinnGen datasets. This mapping may not generalize naturally to other data.

We have now shown that we obtain highly similar mapping when using the eQTL Catalogue and Rahu et al, 2025 datasets (see response to your point 5 above). However, it is certainly possible that on some other datasets the thresholds would be slightly different.

9. In Figures 3D and S2D, the proportion for signals shared between gpu-coloc and CLPP is even higher than CLPP-only. It is not clear whether different denominators were used in these two proportions? Please justify.

We thank the reviewer for this observation. The denominator is the total number of clusters in each bar, not a common reference set. The slightly higher collider rate for the shared subgraph relative to CLPP-only arises because removing CLPP-only edges disproportionately removes small non-collider clusters, so the total cluster count drops faster than the number of collider-containing clusters, raising the rate even as the absolute count of colliders decreases. Following further optimisation of gpu-coloc, we have re-run all analyses, and the two collider rates are now essentially identical (1.118% for shared and 1.124% for CLPP-only, both rounding to 1.12% in Figure 3D). The figures and main text have been updated accordingly.

10. In the section of "Recommendations for large-scale colocalisation analysis", reorganizing the three points may provide better practice guidance. Currently, Point 1 primarily discusses threshold selection for both coloc and CLPP methods. Points 2 and 3 focus on method choice under different data availability scenarios. This seems not a natural order of actions to perform in real data analysis. A more intuitive way may be to first recommend using CLPP when fine-mapping results and credible sets are available and reliable, together with guidance on the appropriate CLPP threshold. And then, when fine-mapping results are unavailable or too noisy, recommend using gpu-coloc, with corresponding guidance on posterior probability thresholds and prior choices.

We agree with the suggested changes. We have now reordered the points and added one additional point that gpu-coloc can also be run using the Torch CPU backend (when GPUs are not available) and still achieve comparable speedup:

1. *If PIPs from fine mapped credible sets are available for all traits of interest, then relying on the CLPP method alone is likely to be the most efficient approach with only minimal loss in sensitivity. Furthermore, fine mapped credible sets for many large-scale GWAS are publicly available from the Open Targets Platform (https://platform.opentargets.org/downloads)[18].*
2. *We recommend using gpu-coloc as a computationally efficient alternative to coloc.bf_bf when either some or all traits have not been fine mapped, or when there is a need to include low-confidence fine mapped signals that fail SuSiE's purity filtering.*
3. *Although our method is called gpu-coloc, it achieves competitive speedup even when only CPUs are available (Figure 2B). Thus, when taking increased cost of GPU hardware into account, using the CPU backend might be the best option for most applications.*
4. *We believe that setting p_{12} prior to 1×10^{-6} and using PP.H4 ≥ 0.8 threshold for gpu-coloc represents a reasonable trade-off between sensitivity and limiting colocalisation colliders for most applications. For the CLPP method, this corresponds to retaining results with CLPP ≥ 0.04.*

Reviewer #3: The paper presents a new implementation of the coloc method that uses GPUs to greatly speed up analyses for very large datasets.
Overall, I think the paper is solid and the analyses are appropriate. In the end, the overlap with the original coloc results is close to perfect, which makes sense given that the underlying mathematics is essentially the same. Any discrepancies may be due to the grouping of loci into large regions based on genomic position, which may substantially enlarge loci and thus affect the final results.
I do have some comments:

1) The paragraph comparing coloc and CLPP is somewhat difficult to follow. I think the language could be made clearer.

2) I am not sure why the authors use CLPP as a "gold standard" when comparing coloc to it. On the one hand, CLPP is essentially a scaled version of PPH4: CLPP is the sum of the product of the two PIPs SNP by SNP, whereas PPH4 is based on the product of the ABFs. However, PIPs are ultimately derived by dividing ABFs by their sum. The key difference is that PPH4 is scaled relative to the evidence for the other hypotheses, while CLPP is not. In this context, I would consider coloc to be more statistically coherent. Therefore, if the goal is to provide recommendations regarding CLPP so that its results are more consistent with coloc, the paragraph should be framed in that way. At the moment, it almost appears to suggest the opposite.

We thank the reviewer for these two insights. We have now made some edits to the paragraph in the introduction that first mentions coloc and CLPP (page 2, lines 39-51):

> *One of the most widely used colocalisation methods is coloc[3] that only requires marginal association summary statistics from both studies. However, coloc makes the restrictive assumption of at most one causal variant per locus. This limitation can be overcome with statistical fine mapping, which distinguishes multiple conditionally distinct signals at the same locus.[10,11] The colocalisation posterior probability (CLPP) method first presented in the eCAVIAR paper performs colocalisation at the level of credible sets, the minimal sets of variants for a fine mapped signal expected to contain the conditionally distinct causal variant with a given probability (e.g. 95%).[4] However, eCAVIAR's exhaustive search fine mapping algorithm made it too slow for most practical datasets.[4,10] Large-scale fine mapping became feasible with the development of the highly scalable FINEMAP[12] and Sum of Single Effects (SuSiE)[13] algorithms. Importantly, both the coloc and CLPP methods have been adopted to support SuSiE output,[6,14] making it possible to use signal-specific Bayes factors (BFs) and credible sets from SuSiE directly in colocalisation.*

We have also rewritten the paragraph comparing posterior probabilities between coloc and CLPP, now explicitly highlighting that while coloc's PP.H4 can be intuitively interpreted at the probability scale (values close to 1 indicate strong evidence for colocalisation), this is not true for CLPP (page 8, lines 309-313):

However, while coloc's PP.H4 has an intuitive interpretation at the locus level (probabilities close to 1 indicate strong evidence for colocalisation), CLPP is a simple sum of variant-level joint probabilities (see Methods) and values as small as 0.1 or even 0.01 are often used as evidence for colocalisation.[4] As a result, it has been challenging to directly compare the colocalisation results from the two methods.[27,28]

3) As the authors state, coloc "colliders" arise from either a lack of power in fine-mapping or insufficient quality control. While coloc should not be applied in the second case, in the first case the correct result would in fact be to detect both colocalizations. Although this situation is a nuisance and difficult to interpret, it is nevertheless a correct result. Therefore, the fact that coloc detects such cases should be considered a positive outcome, as it is closer to the underlying truth.

We agree that colocalisation colliders are not technically false positives *per se*. However, both we and others have found that they can significantly complicate the interpretation of colocalisation results, particularly when the aim is to characterise the pleiotropic consequences of individual causal variants. We have now softened the language and clarified this in main text (page 9, lines 358-363):

While these colocalisation colliders are technically not false positives (there is still at least one shared causal variant in both studies, see example on Figure S3), they complicate the interpretation of conditionally distinct signals which might perturb the target gene via different mechanisms. Thus, when the goal is to characterise the pleiotropic impact of individual causal variants[18,30], it might be desirable to reduce colocalisation colliders as much as possible.

4) The paragraph comparing colocalization with and without fine-mapping does not add much, as there is no clear reason to expect results that differ from what is already known.

We agree that these results are not particularly novel. However, in parallel work (https://elixir.ut.ee/eqtl/about-nmr-coloc), we have found the gpu-coloc to be most useful when assessing colocalisation with GWAS datasets where reliable fine-mapping results are not available, such as the Pan-UKBB resource (https://pan.ukbb.broadinstitute.org/) or the FinnGen + UK Biobank + Million Veterans Program GWAS meta-analysis (https://public-mvp-ukbb.finngen.fi/). Thus, we believe that it is informative for the prospective users of gpu-coloc to know what the expected performance is.

If the authors would like to frame the paper as a guide on how to run and properly interpret colocalization analyses (which could be a valid option), more work would be needed to explore the impact of various parameters, perhaps using simulations. However, this may be beyond the scope of the current study.

We thank the reviewer for this suggestion and agree that a thorough parameter exploration would be valuable, but we feel it falls outside the scope of the current paper.
