# Supplementary material for: Ultra-fast genetic colocalisation across millions of association signals
Source: PLoS Genet. 2026 Jun 17;22(6):e1012209. doi: 10.1371/journal.pgen.1012209 (PMC13289940; doi:10.1371/journal.pgen.1012209)
Supplement: Peer review history

# Peer review: Decision Letter 0

**DOI:** 10.1371/journal.pgen.1012209.r001

**Authors:** Michael P. Epstein, Lin S. Chen

2 Mar 2026

PGENETICS-D-26-00069

Ultra-fast genetic colocalisation across millions of traits

PLOS Genetics

Dear Dr. Alasoo,

Thank you for submitting your manuscript to PLOS Genetics. The editorial board and three reviewers have carefully evaluated the manuscript and believe it has potential value, but it does not fully meet PLOS Genetics's publication criteria as it currently stands. Therefore, we invite you to submit a revised version of the manuscript that addresses the points raised by the reviewers.

Please submit your revised manuscript by May 01 2026 11:59PM. If you will need more time than this to complete your revisions, please reply to this message or contact the journal office at plosgenetics@plos.org. When you're ready to submit your revision, log on to https://www.editorialmanager.com/pgenetics/ and select the 'Submissions Needing Revision' folder to locate your manuscript file.

Please include the following items when submitting your revised manuscript:

* A letter that responds to each point raised by the editor and reviewer(s). You should upload this letter as a separate file labeled 'Response to Reviewers'. This file does not need to include responses to any formatting updates and technical items listed in the 'Journal Requirements' section below.

* A marked-up copy of your manuscript that highlights changes made to the original version. You should upload this as a separate file labeled 'Revised Manuscript with Track Changes'.

* An unmarked version of your revised paper without tracked changes. You should upload this as a separate file labeled 'Manuscript'.

If you would like to make changes to your financial disclosure, competing interests statement, or data availability statement, please make these updates within the submission form at the time of resubmission. Guidelines for resubmitting your figure files are available below the reviewer comments at the end of this letter.

We look forward to receiving your revised manuscript.

Kind regards,

Lin S. Chen, Ph.D.

Academic Editor

PLOS Genetics

Michael P. Epstein

Section Editor

PLOS Genetics

Aimée Dudley

Editor-in-Chief

PLOS Genetics

Anne Goriely

Editor-in-Chief

PLOS Genetics

**Journal Requirements:**

1) We ask that a manuscript source file is provided at Revision. Please upload your manuscript file as a .doc, .docx, .rtf or .tex. If you are providing a .tex file, please upload it under the item type u2018LaTeX Source Fileu2019 and leave your .pdf version as the item type u2018Manuscriptu2019.

2) Please provide an Author Summary. This should appear in your manuscript between the Abstract (if applicable) and the Introduction, and should be 150-200 words long. The aim should be to make your findings accessible to a wide audience that includes both scientists and non-scientists. Sample summaries can be found on our website under Submission Guidelines:

https://journals.plos.org/plosgenetics/s/submission-guidelines#loc-parts-of-a-submission

3) Please upload all main figures as separate Figure files in .tif or .eps format. For more information about how to convert and format your figure files please see our guidelines:

https://journals.plos.org/plosgenetics/s/figures

4) We have noticed that you have uploaded Supporting Information files, but you have not included a complete list of legends. Please add a full list of legends for your Supporting Information file (supplementary table) after the references list.

5) We notice that your supplementary Figures are included in the manuscript file. Please remove them and upload them with the file type 'Supporting Information'. Please ensure that each Supporting Information file has a legend listed in the manuscript after the references list.

6) Please amend your detailed Financial Disclosure statement. This is published with the article. It must therefore be completed in full sentences and contain the exact wording you wish to be published.

1) State what role the funders took in the study. If the funders had no role in your study, please state: "The funders had no role in study design, data collection and analysis, decision to publish, or preparation of the manuscript."

2) If any authors received a salary from any of your funders, please state which authors and which funders..

Note: If the reviewer comments include a recommendation to cite specific previously published works, please review and evaluate these publications to determine whether they are relevant and should be cited. There is no requirement to cite these works unless the editor has indicated otherwise.

**Reviewers' comments:**

Reviewer's Responses to Questions**.**

Reviewer #1: The authors introduced gpu-coloc, a GPU-accelerated re-implementation of the coloc algorithm that enables biobank-scale colocalisation. Through comprehensive computational benchmarking, they demonstrated the efficiency of gpu-coloc. They also investigated the colocalisation colliders and provided practical recommendations in real data analysis for users. I have some comments as follows.

1. Although the authors explained PP.H4 in detail in the Methods section, it would be helpful to briefly introduce its meaning when it is first mentioned. This would make the manuscript more accessible to a more general audience.

2. Line 153-156: Why is the gpu-coloc PP.H4 considered more conservative than that from coloc.bf_bf? According to Figure 2A, the PP.H4 values between the two methods are virtually identical, with only a few outliers where gpu-coloc reports lower values. Notably, these discrepancies are concentrated in the region where PP.H4 < 0.6, which may have limited impact on the false positive rate in practice. Furthermore, I don't think that being more conservative is necessarily superior.

3. Line 164-174: The major contribution of this paper lies in its highly efficient implementation. Therefore, I suggest providing more detailed information when benchmarking the computation time, such as the hardware of the high-performance computing system (GPU model, CPU model, number of cores, RAM), computation configuration (number of parallel processes), and the time spent on model fitting versus I/O operations. Additionally, how does the computation time of gpu-coloc scale with the dataset size? Identifying the primary computational bottleneck would be highly beneficial for potential users.

4. How does the computational efficiency of gpu-coloc compare with other popular efficient methods (e.g., HyPrColoc, https://doi.org/10.1038/s41467-020-20885-8)? I suggest including more competing methods for a more comprehensive comparison.

5. The empirical strategy to select the p12 prior and corresponding CLPP thresholds by quantifying the rate of colocalisation colliders is very interesting and valuable to users. However, relying solely on this metric may be limiting since the colocalisation colliders are not strictly equivalent to false positives. Have the authors considered other metrics?

6. It's interesting to see that the SuSiE signals that failed purity filtering may cause the increased rate of colocalisation colliders for gpu-coloc. In the real data analyses, did the authors observe any concrete examples of such collider events? For example, do these signals exhibit broader or multi-modal PIP distributions, or more complex LD structures that prevent them from passing the purity filtering?

7. Figure 3C: Does CS refer to credible set? I suggest explaining the abbreviation at first use.

Reviewer #2: In this manuscript, the authors introduced a scalable implementation of colocalization algorithm. They compared locus-level posteriors with variant-level overlap metrics such as CLPP and provided practical recommendations for choosing pipelines, thresholds, and priors in colocalization analyses. The work is likely to be impactful for the statistical genetics community, particularly for large-scale integrative analyses involving GWAS and molecular QTL datasets. However, several aspects of the study would benefit from clearer explanation, including implementation choices for handling missing values, the design of benchmarking, and parts of the practical recommendations. Detailed comments are provided below:

1. In the title, "across millions of traits" is potentially misleading. As presented in the following analyses, it appear to involve millions of colocalization tests or locus-trait or signal-trait pairs, rather than millions of distinct traits. Rephrasing the title to more precisely reflect the scale of the analyses would improve clarity.

2. The workaround of replacing missing LBF values with an extreme negative constant is a critical implementation step that enables the matrix-based computations in gpu-coloc. To improve transparency, It would be helpful to explicitly illustrate this step in Figure 1. In addition, please justify the choice of the value of -1e6. It is unclear whether this threshold should remain fixed across different datasets or analysis settings, or whether it should depend on factors such as sample size, effect size distributions, or prior choices. A brief sensitivity analysis assessing the robustness of posterior inferences to the choice of this constant would further strengthen confidence in the method.

3. The gpu-coloc replaces missing LBFs with an extreme negative value, so the difference in PP.H4 shown in Figure 2A may depend on the pattern and extent of missingness across datasets, traits, locus, and variants. Please clarify how frequently the replacement occurs in practice, and whether heterogeneity in missingness could influence the observed differences between methods.

4. The results of computational performance on pages 5-6 would benefit from additional clarification and reorganization. (1) When reporting the running times, it would be helpful to clearly specify the computational settings, including allocated memory, number of cores, and the total number of colocalization tests performed. Reporting a normalized metric such as running time per test may facilitate more meaningful comparison across methods and computing environments. (2) The manuscript mentions running times on a personal Mac computer in both lines 168 and 179, which appear to convey similar messages regarding computational efficiency. Reorganizing these descriptions could improve clarity and avoid redundancy. (3) Finally, to better demonstrate scalability, it would be informative to show how running time scales with the number of colocalization tests, and to compare with existing methods.

5. The gpu-coloc is compared with coloc.bf_bf and with CLPP using different datasets. It would be helpful for the authors to clarify the rationale and criterion in choosing different datasets for different comparisons. In particular, please explain whether this choice reflects method-specific input requirements, data availability, or distinct benchmarking goals. Without such clarification, it is difficult to disentangle method differences from dataset-specific effects.

7. From "we leveraged our efficient gpu-coloc implementation to calculate both the CLPP and PP.H4 values", it seems the gpu-coloc method can be used as re-implementation for both standard coloc and CLPP. But in the method overview and Figure 1, it seems gpu-coloc is only a more efficient tool for standard coloc. Please clarify.

8. The mapping between CLPP and coloc posterior probability (page 6-7) is derived empirically using the eQTL Catalogue and FinnGen datasets. This mapping may not generalize naturally to other data.

9. In Figures 3D and S2D, the proportion for signals shared between gpu-coloc and CLPP is even higher than CLPP-only. It is not clear whether different denominators were used in these two proportions? Please justify.

10. In the section of "Recommendations for large-scale colocalisation analysis", reorganizing the three points may provide better practice guidance. Currently, Point 1 primarily discusses threshold selection for both coloc and CLPP methods. Points 2 and 3 focus on method choice under different data availability scenarios. This seems not a natural order of actions to perform in real data analysis. A more intuitive way may be to first recommend using CLPP when fine-mapping results and credible sets are available and reliable, together with guidance on the appropriate CLPP threshold. And then, when fine-mapping results are unavailable or too noisy, recommend using gpu-coloc, with corresponding guidance on posterior probability thresholds and prior choices.

Reviewer #3: The paper presents a new implementation of the coloc method that uses GPUs to greatly speed up analyses for very large datasets.

Overall, I think the paper is solid and the analyses are appropriate. In the end, the overlap with the original coloc results is close to perfect, which makes sense given that the underlying mathematics is essentially the same. Any discrepancies may be due to the grouping of loci into large regions based on genomic position, which may substantially enlarge loci and thus affect the final results.

I do have some comments:

1) The paragraph comparing coloc and CLPP is somewhat difficult to follow. I think the language could be made clearer.

2) I am not sure why the authors use CLPP as a “gold standard” when comparing coloc to it. On the one hand, CLPP is essentially a scaled version of PPH4: CLPP is the sum of the product of the two PIPs SNP by SNP, whereas PPH4 is based on the product of the ABFs. However, PIPs are ultimately derived by dividing ABFs by their sum. The key difference is that PPH4 is scaled relative to the evidence for the other hypotheses, while CLPP is not. In this context, I would consider coloc to be more statistically coherent. Therefore, if the goal is to provide recommendations regarding CLPP so that its results are more consistent with coloc, the paragraph should be framed in that way. At the moment, it almost appears to suggest the opposite.

3) As the authors state, coloc “colliders” arise from either a lack of power in fine-mapping or insufficient quality control. While coloc should not be applied in the second case, in the first case the correct result would in fact be to detect both colocalizations. Although this situation is a nuisance and difficult to interpret, it is nevertheless a correct result. Therefore, the fact that coloc detects such cases should be considered a positive outcome, as it is closer to the underlying truth.

4) The paragraph comparing colocalization with and without fine-mapping does not add much, as there is no clear reason to expect results that differ from what is already known.

If the authors would like to frame the paper as a guide on how to run and properly interpret colocalization analyses (which could be a valid option), more work would be needed to explore the impact of various parameters, perhaps using simulations. However, this may be beyond the scope of the current study.

**********

**Have all data underlying the figures and results presented in the manuscript been provided?**

Large-scale datasets should be made available via a public repository as described in the *PLOS Genetics*   data availability policy, and numerical data that underlies graphs or summary statistics should be provided in spreadsheet form as supporting information.

Reviewer #1: None

Reviewer #2: None

Reviewer #3: Yes

**********

PLOS authors have the option to publish the peer review history of their article (what does this mean?). If published, this will include your full peer review and any attached files.

If you choose “no”, your identity will remain anonymous but your review may still be made public.

**Do you want your identity to be public for this peer review?**   For information about this choice, including consent withdrawal, please see our Privacy Policy.

Reviewer #1: No

Reviewer #2: No

Reviewer #3: No

[NOTE: If reviewer comments were submitted as an attachment file, they will be attached to this email and accessible via the submission site. Please log into your account, locate the manuscript record, and check for the action link "View Attachments". If this link does not appear, there are no attachment files.]

**Figure resubmission:**

While revising your submission, we strongly recommend that you use PLOS’s NAAS tool (https://ngplosjournals.pagemajik.ai/artanalysis) to test your figure files. NAAS can convert your figure files to the TIFF file type and meet basic requirements (such as print size, resolution), or provide you with a report on issues that do not meet our requirements and that NAAS cannot fix.

After uploading your figures to PLOS’s NAAS tool - https://ngplosjournals.pagemajik.ai/artanalysis, NAAS will process the files provided and display the results in the "Uploaded Files" section of the page as the processing is complete. If the uploaded figures meet our requirements (or NAAS is able to fix the files to meet our requirements), the figure will be marked as "fixed" above. If NAAS is unable to fix the files, a red "failed" label will appear above. When NAAS has confirmed that the figure files meet our requirements, please download the file via the download option, and include these NAAS processed figure files when submitting your revised manuscript.

**Reproducibility:**

To enhance the reproducibility of your results, we recommend that authors of applicable studies deposit laboratory protocols in protocols.io, where a protocol can be assigned its own identifier (DOI) such that it can be cited independently in the future. Additionally, PLOS ONE offers an option to publish peer-reviewed clinical study protocols. Read more information on sharing protocols at https://plos.org/protocols?utm_medium=editorial-email&utm_source=authorletters&utm_campaign=protocols

# Author comment: Author response to Decision Letter 1

**DOI:** 10.1371/journal.pgen.1012209.r002

9 May 2026

Attachment Submitted filename: Response to reviewers.pdf

# Peer review: Decision Letter 1

**DOI:** 10.1371/journal.pgen.1012209.r003

**Authors:** Michael P. Epstein, Lin S. Chen

7 Jun 2026

Dear Dr Alasoo,

We are pleased to inform you that your manuscript entitled "Ultra-fast genetic colocalisation across millions of association signals" has been editorially accepted for publication in PLOS Genetics. Congratulations!

Before your submission can be formally accepted and sent to production you will need to complete our formatting changes, which you will receive in a follow up email. Please be aware that it may take several days for you to receive this email; during this time no action is required by you. Please note: the accept date on your published article will reflect the date of this provisional acceptance, but your manuscript will not be scheduled for publication until the required changes have been made.

Once your paper is formally accepted, an uncorrected proof of your manuscript will be published online ahead of the final version, unless you’ve already opted out via the online submission form. If, for any reason, you do not want an earlier version of your manuscript published online or are unsure if you have already indicated as such, please let the journal staff know immediately at plosgenetics@plos.org.

In the meantime, please log into Editorial Manager at https://www.editorialmanager.com/pgenetics/, click the "Update My Information" link at the top of the page, and update your user information to ensure an efficient production and billing process. Note that PLOS requires an ORCID iD for all corresponding authors. Therefore, please ensure that you have an ORCID iD and that it is validated in Editorial Manager. To do this, go to ‘Update my Information’ (in the upper left-hand corner of the main menu), and click on the Fetch/Validate link next to the ORCID field.  This will take you to the ORCID site and allow you to create a new iD or authenticate a pre-existing iD in Editorial Manager.

If you have a press-related query, or would like to know about making your underlying data available (as you will be aware, this is required for publication), please see the end of this email. If your institution or institutions have a press office, please notify them about your upcoming article at this point, to enable them to help maximise its impact. Inform journal staff as soon as possible if you are preparing a press release for your article and need a publication date.

Thank you again for supporting open-access publishing; we are looking forward to publishing your work in PLOS Genetics!

Yours sincerely,

Lin S. Chen, Ph.D.

Academic Editor

PLOS Genetics

Michael Epstein

Section Editor

PLOS Genetics

Aimée Dudley

Editor-in-Chief

PLOS Genetics

Anne Goriely

Editor-in-Chief

PLOS Genetics

www.plosgenetics.org

BlueSky: @plos.bsky.social

----------------------------------------------------

Comments from the reviewers (if applicable):

Reviewer's Responses to Questions

**Comments to the Authors:**

**Please note here if the review is uploaded as an attachment.**

Reviewer #1: The authors have addressed all of my concerns.

Reviewer #2: The authors have addressed all my comments.

Reviewer #3: I have no further comments

**********

**Have all data underlying the figures and results presented in the manuscript been provided?**

Large-scale datasets should be made available via a public repository as described in the *PLOS Genetics*   data availability policy, and numerical data that underlies graphs or summary statistics should be provided in spreadsheet form as supporting information.

Reviewer #1: Yes

Reviewer #2: None

Reviewer #3: None

**********

PLOS authors have the option to publish the peer review history of their article (what does this mean?). If published, this will include your full peer review and any attached files.

If you choose “no”, your identity will remain anonymous but your review may still be made public.

**Do you want your identity to be public for this peer review?**   For information about this choice, including consent withdrawal, please see our Privacy Policy.

Reviewer #1: No

Reviewer #2: No

Reviewer #3: No

----------------------------------------------------

**Data Deposition**

If you have submitted a Research Article or Front Matter that has associated data that are not suitable for deposition in a subject-specific public repository (such as GenBank or ArrayExpress), one way to make that data available is to deposit it in the Dryad Digital Repository. As you may recall, we ask all authors to agree to make data available; this is one way to achieve that. A full list of recommended repositories can be found on our website.

The following link will take you to the Dryad record for your article, so you won't have to re‐enter its bibliographic information, and can upload your files directly:

http://datadryad.org/submit?journalID=pgenetics&manu=PGENETICS-D-26-00069R1

More information about depositing data in Dryad is available at http://www.datadryad.org/depositing. If you experience any difficulties in submitting your data, please contact help@datadryad.org for support.

Additionally, please be aware that our data availability policy requires that all numerical data underlying display items are included with the submission, and you will need to provide this before we can formally accept your manuscript, if not already present.

----------------------------------------------------

**Press Queries**

If you or your institution will be preparing press materials for this manuscript, or if you need to know your paper's publication date for media purposes, please inform the journal staff as soon as possible so that your submission can be scheduled accordingly. Your manuscript will remain under a strict press embargo until the publication date and time. This means an early version of your manuscript will not be published ahead of your final version. PLOS Genetics may also choose to issue a press release for your article. If there's anything the journal should know or you'd like more information, please get in touch via plosgenetics@plos.org.

# Editor report: Acceptance letter

**DOI:** 10.1371/journal.pgen.1012209.r004

**Authors:** Michael P. Epstein, Lin S. Chen

PGENETICS-D-26-00069R1

Ultra-fast genetic colocalisation across millions of association signals

Dear Dr Alasoo,

We are pleased to inform you that your manuscript entitled "Ultra-fast genetic colocalisation across millions of association signals" has been formally accepted for publication in PLOS Genetics! Your manuscript is now with our production department and you will be notified of the publication date in due course.

The corresponding author will soon be receiving a typeset proof for review, to ensure errors have not been introduced during production. Please review the PDF proof of your manuscript carefully, as this is the last chance to correct any errors. Please note that major changes, or those which affect the scientific understanding of the work, will likely cause delays to the publication date of your manuscript.

Soon after your final files are uploaded, unless you have opted out or your manuscript is a front-matter piece, the early version of your manuscript will be published online. The date of the early version will be your article's publication date. The final article will be published to the same URL, and all versions of the paper will be accessible to readers.

For Research Articles, you will receive an invoice from PLOS for your publication fee after your manuscript has reached the completed accept phase. If you receive an email requesting payment before acceptance or for any other service, this may be a phishing scheme. Learn how to identify phishing emails and protect your accounts at https://explore.plos.org/phishing.

Thank you again for supporting PLOS Genetics and open-access publishing. We are looking forward to publishing your work!

With kind regards,

Zsofia Freund

PLOS Genetics

On behalf of:

The PLOS Genetics Team

Carlyle House, Carlyle Road, Cambridge CB4 3DN | United Kingdom

plosgenetics@plos.org | +44 (0) 1223-442823

plosgenetics.org | Twitter: @PLOSGenetics
